# Characteristics of ante- and postnatal risk factors of the food allergy in infants

**DOI:** 10.1186/2045-7022-3-S3-P110

**Published:** 2013-07-25

**Authors:** SN Denisova, MY Belitskaya, TB Sentsova, VA Revyakina, EV Pavlovskaya, AA Trofimova

**Affiliations:** 1 Russian National Research Medical University, Moscow, Russian Federation; 2 Research Institute of Nutrition, Russian Academy of Medical Sciences, Moscow, Russian Federation

## Background

Risk factors which promote the development of allergy to the cow milk protein in infants include antenatal sensitization, bottle feeding of neonates, early start of bottle or mixed feeding, postnatal sensitization of mite and pollen allergy. The aim of the study was to analyze a family history of the allergic diseases, course of pregnancy and delivery in mothers, effect of concomitant pathology on the development and course of food allergy in infants.

## Methods

We examined 471 infants with food allergy and several degrees of the atopic dermatitis (AD). Moderate and severe AD were more common at the age of 0-12 months old than at the age of 12-36 months old (80.9% and 55.6%, respectively). In boys of the 12-36 months old severe course of the disease was more common than in girls of the same age (14.6% and 7.3%, respectively). We used SCORAD index for estimation of AD severity.

## Results

Positive family histories of the allergic diseases were revealed in 67.9% and 67.3% infants with moderate and severe course of AD, respectively. In the half of these infants allergic diseases were found in the maternal line. Disturbances of the pregnancy and delivery in mothers of the infants with and without AD were equal; however these disturbances were more common in mothers of the infants with severe AD. The postnatal risk factors of allergy were revealed in 11.7% of infants: prematurity (3.2%), prenatal hypotrophy (4.7%), intranatal asphyxia (2.3%), cephalohaematoma (1.5%).

## Conclusion

The positive family history of allergy was high in all infants with AD independently on the age of the appearance of first signs of food allergy. Mothers with pathological course of pregnancy and delivery more often had infants with severe course of AD. Prevalence of the postnatal risk factors in healthy and ill infants was equal.

## Disclosure of interest

None declared.

